# Indomethacin Fails to Increase Intestinal Permeability in Healthy Volunteers

**DOI:** 10.14309/ctg.0000000000000944

**Published:** 2025-10-28

**Authors:** Michael Camilleri, Irene Busciglio, Paula Carlson, Saam Dilmaghani, Camille Lupianez-Merly, David Y. Yang, Michael Ryks, Monique Ferber, Dounia Houamel, Stéphanie Perot, François Montestruc

**Affiliations:** 1 Clinical Enteric Neuroscience and Translational Epidemiological Research (CENTER), Mayo Clinic, Rochester, Minnesota, USA;; 2 Biocodex R&D Center, Compiègne, France;; 3 eXYSTAT, Malakoff, France.

**Keywords:** intestinal permeability, indomethacin, gut barrier

## Abstract

**INTRODUCTION:**

Indomethacin is often used experimentally to induce intestinal hyperpermeability, enabling evaluation of interventions targeting barrier function.

**METHODS:**

We conducted a randomized, double-blind, placebo-controlled study (NCT05538247) in healthy volunteers to assess whether a supplement could mitigate indomethacin-induced hyperpermeability. Participants received 150 mg/d of indomethacin for 6 days, either before or during placebo/supplement administration. Permeability was measured using ^13^C-mannitol and lactulose urinary excretion.

**RESULTS:**

Contrary to expectations, indomethacin failed to increase ^13^C-mannitol excretion in either group. No meaningful elevations in serum (zonulin, claudins) or fecal (calprotectin) biomarkers were observed.

**DISCUSSION:**

Our findings suggest that the expected increase in intestinal permeability after indomethacin administration may not be consistently observed in healthy volunteers. These results highlight the need to carefully consider the reproducibility and sensitivity of this model in future clinical studies aiming to investigate gut barrier function.

## INTRODUCTION

Inducing intestinal barrier dysfunction with nonsteroidal anti-inflammatory drugs (NSAIDs) such as indomethacin has been a common approach to explore intestinal permeability (1,2) and assess interventions designed to restore barrier integrity. The aim of our study was to investigate whether an oral supplement could strengthen the intestinal barrier function and counteract the acute hyperpermeability induced by the NSAID, indomethacin, in healthy human volunteers. In this report, we have assessed the effects of exogenous administration of indomethacin in healthy human volunteers.

## METHODS

**Figure 1.**
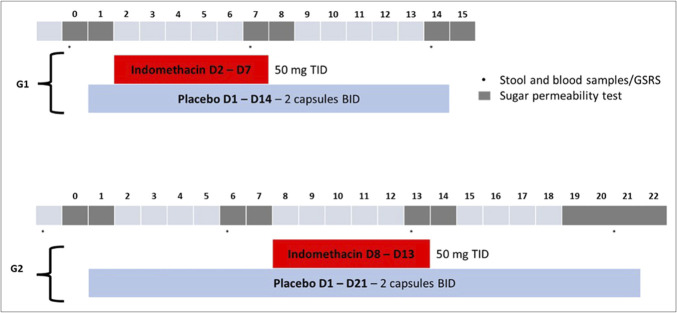
Experimental protocol showing days of administration of placebo and indomethacin. bid, twice a day; tid, 3 times a day; D, day; G1, Group 1; G2, Group 2; GSRS, gastrointestinal symptoms rating scale.

This was a randomized, double-blind, placebo-controlled, parallel-arm clinical study (NCT05538247) in healthy adult volunteers. The study was divided into 2 separate substudies referred to as Group 1 and Group 2, in which the initiation and duration of the study products differed according to the group. Subjects could freely choose to participate in Group 1 or Group 2 at the screening visit, according to their availability. At the inclusion visit, in each group, eligible subjects were randomized (1:1) in a double-blind manner to receive either experimental therapy or placebo. All Group 1 and Group 2 subjects received an open-label, short-term (6 days) indomethacin challenge (150 mg/d) to increase intestinal permeability: For Group 1 subjects, treatment was started with indomethacin and lasted 14 days; for Group 2 subjects, treatment was started 7 days before indomethacin and lasted 20(±1) days (Figure 1).

### Measurement of intestinal permeability and endpoints

The permeability test was based on the excretion of ingested sugar probes (1,000 mg lactulose and 100 mg ^13^C-mannitol) in accordance with a previously validated assay (3) in distinct urine collection intervals to selectively characterize the permeability of different parts of the gastrointestinal tract: The 0–2-hour urine collection was a marker of small intestinal permeability, the 2–8-hour urine collection was a marker of both small intestinal and colonic permeability, and the 8–24-hour urine collection was a marker of colonic permeability. The primary permeability end point in the analysis was ^13^C-mannitol excretion 2–24 hours (mg) expressed as the change from baseline to the end of treatment with indomethacin, that was day 7 in Group 1 and day 13 in Group 2.

Additional secondary observations included fecal and serum biomarkers of barrier function and intestinal injury [calprotectin (4), zonulin (5), claudin 1 (6), C-reactive protein (CRP) (7)] and symptoms scores (gastrointestinal symptom rating scale [GSRS]).

### Gastrointestinal symptom response scale

The GSRS is a validated questionnaire to evaluate gastrointestinal symptoms in gastrointestinal disorders (8).

### Statistical analysis

The primary end point was absolute change from baseline of ^13^C-mannitol 2–24 hours (mg) assessed with an analysis of covariance model which included terms to adjust for the baseline value and treatment arm. The average of the adjusted difference in the mean absolute change from baseline was reported with SEs of the mean, 2-sided 95% confidence interval (CI), and *P* value. Multiple imputation using the fully conditional specification method was used for missing data.

## RESULTS

Supplement Table 1 (http://links.lww.com/CTG/B415) presents demographics, participant habits, bowel function, significant medical history at baseline, and compliance with protocol treatment of all participants in group 1 and group 2 of study. There were no significant differences among the 2 groups.

Table 1 summarizes the permeability end point analyses, based on the primary urinary excretion of ^13^C-mannitol 2–24 hours (mg) and secondary end points of urinary excretion of lactulose 2–24 hours (mg), as well as serum biomarkers of intestinal injury and intestinal permeability. None of these changes were statistically significant at the 5% level.

**Table 1.** Permeability end point analyses and serum or fecal biomarkers of intestinal injury and intestinal permeability

GROUP 1: 7 d indomethacin with concurrent placebo			
	Baseline	Day 7 indomethacin	Change from baseline to Day 7
^13^C-Mannitol at 2–24 hr (mg)			
N	13	13	13
Mean (SD); [95% CI of mean]	7.0 (4.8); [4.1; 9.9]	6.6 (3.4); [4.5; 8.7]	−0.4 (4.9); [−3.3; 2.6]
Median	5.2	6.1	1.2
Lactulose at 2–24 hr (mg)			
N	13	13	13
Mean (SD); [95% CI of mean]	1.8 (1.2) [1.1; 2.5]	2.6 (2.0) [1.4; 3.9]	0.8 (1.6); [−0.1; 1.8]
Median	1.4	1.6	0.0

GROUP 2: 7 d indomethacin with 6 day prior and concurrent placebo			
	Baseline	Day 13 indomethacin	Change from baseline to Day 13
N	12	11	11
Mean (SD); [95% CI of mean]	12.1 (7.4); [7.3; 16.8]	8.1 (4.7); [5.0; 11.3]	−4.7 (9.5); [−11.1; 1.7]
Median	10.6	7.3	−2.5
Lactulose at 2–24 hr (mg)			
N	12	11	11
Mean (SD); [95% CI of mean]	2.6 (1.7); [1.6; 3.7]	2.3 (1.9); [1.1; 3.6}	−0.5 (2.2); [−2.0; 1.0]
Median	2.0	1.9	−0.0
Change from baseline to D7 (G1) or D13 (G2) for serum and fecal markers: Mean (SD); [95% CI of mean]			
Serum zonulin ng/mL G1	35.4 (10.6); [27.3; 43.6]	38.0 (7.4); [31.8; 44.2]	2.6 (7.9); [−4.7; 9.8]
Serum zonulin ng/mL G2	35.5 (9.4); [29.2; 41.9]	35.4 (7.5); [30.6; 40.2]	−0.3 (8.4) [−6.0; 5.4]
Serum claudin 1 ng/mL G1	2.8 (0.7); [2.4; 3.3]	2.8 (0.9); [2.3; 3.4]	−0.1 (0.7); [−0.5; 0.4]
Serum claudin 1 ng/mL G2	2.4 (0.9); [1.8; 2.9]	2.7 (1.2); [2.0; 3.4]	0.4 (1.0); [−0.3; 1.1]
Serum CRP (mg/dL) G1	0.1 (0.1); [0.0; 0.2]	0.1 (0.1); [0.0; 0.2]	−0.0 (0.1); [−0.1; 0.0]
Serum CRP (mg/dL) G2	0.2 (0.2); [0.1; 0.4]	0.3 (0.3); [0.1; 0.5]	0.1 (0.2); [−0.1; 0.2]
Fecal calprotectin G1	15.1 (9.9); [9.2; 21.1]	28.5 (41.1); [2.4; 54.6]	15.7 (36.5); [−7.4; 38.9]
Fecal calprotectin G2	11.7 (1.8); [10.6; 12.9]	36.8 (46.9); [5.3; 68.4]	25.0 (47.3); [−6.7; 56.8]
Clinical effects assessed by abdominal pain score by gastrointestinal symptoms rating scale			
Total score G1	15.5 (1.1); [14.8; 16.1]	17.3 (3.0); [15.5; 19.1]	1.8 (2.1); [0.6; 3.1]
Total score G2	15.9 (1.1); [15.2; 16.6]	18.8 (5.9); [15.1; 22.6]	2.9 (5.7); [−0.7; 6.5]
Abdominal pain score G1	3.0 (0.0)	3.7 (0.9); [3.1; 4.3]	0.7 (0.9); [0.1; 1.3]
Abdominal pain score G2	3.3 (0.5); [3.0; 3.5]	3.8 (1.3); [2.9; 4.6]	0.5 (1.2); [−0.3; 1.3]
Diarrhea score G1	3.0 (0.0)	3.5 (0.9); [2.9; 4.0]	0.5 (0.9); [−0.1; 1.0]
Diarrhea score G2	3.0 (0.0)	4.9 (3.7); [2.6; 7.3]	1.9 (3.7); [−0.4; 4.3]
Indigestion score G1	4.2 (0.6); [3.9; 4.6]	4.8 (1.5); [3.9; 5.8]	0.6 (1.0); [−0.0; 1.2]
Indigestion score G2	4.3 (0.5); [4.0; 4.6]	4.7 (1.2); [3.9; 5.4]	0.3 (1.0); [−0.3; 1.0]

### Change in ^13^C-mannitol 2–24 hours from baseline to day 7 in Group 1 and to day 13 in Group 2

In the placebo arm, mean values of ^13^C-mannitol 2–24 hours were similar between baseline and day 7, with an adjusted mean change from baseline (95% CI) of −0.5 mg [−4.9; 3.8]. Similarly, in the placebo arm, mean values of ^13^C-mannitol 2–24 hours were similar between baseline and day 13, with an adjusted mean change from baseline (95% CI) of −3.7 mg [−7.6; 0.1]. The lack of increase in excretion of ^13^C-mannitol 2–24 hours suggests that indomethacin did not increase intestinal permeability in subjects treated with placebo in either group.

### Changes in secondary endpoints

No meaningful changes were observed in serum zonulin or claudins, nor in fecal calprotectin. GSRS total scores increased modestly, without corresponding biomarker shifts.

### Gastrointestinal symptoms

Based on the total score and abdominal pain score on the GSRS, there was a significant increase in total score and abdominal pain score in subjects in Group 1 but not in Group 2. Note also the increase in diarrhea score in Group 2, which was associated with large variance and did not reach statistical significance.

## DISCUSSION

Although indomethacin was associated with adverse effects, it is worth noting that several biomarkers used to assess changes in intestinal permeability using oral sugar probe molecules, and using serum and fecal measurements did not identify such effects of indomethacin on intestinal permeability. These results do not support a consistent increase in intestinal permeability after indomethacin administration. Despite its frequent use, our findings indicate that indomethacin may not consistently provoke intestinal hyperpermeability in healthy individuals, as measured by sugar probe excretion or biomarker analysis. It is important to keep this in perspective in relation with future studies addressing the potential effects on intestinal barrier function of novel experimental therapeutic approaches. Future research may consider higher NSAID dose, longer exposure periods, or study populations exhibiting preexisting gut barrier alteration.

## CONFLICTS OF INTEREST

**Guarantor of the article:** Michael Camilleri, MD, DSc, MACG.

**Specific author contributions:** M.C.: PI, senior author, conception of project, writing and revising manuscript. I.B.: laboratory supervisor. P.C.: study coordinator. S.D., C.L.-M., and D.Y.Y.: participant care, revising manuscript. M.R.: laboratory technician. M.F.: study coordinator. D.H.: study protocol development, revising manuscript. S.P., and F.M.: statistician, revising manuscript. 

**Financial support:** This study was funded by a research grant from Biocodex, 7 avenue Gallieni, 94250 Gentilly, France.

**Potential competing interests:** Dounia Houamel is an employee of Biocodex. The other authors have no conflicts of interest.

**Trial registry:** ClinicalTrials.gov #NCT05538247.

## Supplementary Material
