# Supplementary material for: Indomethacin Fails to Increase Intestinal Permeability in Healthy Volunteers
Source: Clin Transl Gastroenterol. 2025 Oct 28;17(1):e00944. doi: 10.14309/ctg.0000000000000944 (PMC12818839; doi:10.14309/ctg.0000000000000944)
Supplement: Supplementary file 1 [file ct9-17-e00944-s001.docx]

**SUPPLEMENT MATERIAL – Table 1. Demographics, participant habits, bowel function, significant medical history at baseline, as well as compliance with protocol treatment of all participants in group 1 and group 2 of study. Data show median [IQR], or N (%).**

| **Parameter** | **Group 1** | **Group 2** | **Both groups** |
| --- | --- | --- | --- |
| **N** | 13 | 12 | 25 |
| **Age (years)** *Median - Q1 - Q3* | 30.0 [27.0 - 40.0] | 28.5 [26.0 - 35.5] | 30.0 [26.0 - 36.0] |
| **Female: male (%)** | 38.5%: 61.5% | 58.3%: 41.7% | 48.0%: 52.0% |
| **Height (cm)** *Median - Q1 - Q3* | 170.8 [162.5 - 177.5] | 168.8 [160.5 - 179.0] | 170.8 [162.5 - 177.5] |
| **Weight (kg)** *Median - Q1 - Q3* | 77.2 [64.0 - 90.6] | 69.0 [62.8 - 86.9] | 74.4 [64.0 - 87.6] |
| **BMI (kg/m^2^)** *Median - Q1 - Q3* | 26.6 [25.2 - 29.3] | 24.0 [22.6 - 28.4] | 25.8 [23.3 - 29.1] |
| **Non-smoker or former smoker** | 13 (100%) | 12 (100%) | 25 (100%) |
| **Special dietary requirements** | 2/13 (type of meat exclusion) | 0/12 | 2/25 (type of meat exclusion) |
| **Lactose-free diet** | 0 | 0 | 0 |
| **Participant in intensive sports** | 0 | 0 | 0 |
| **Bowel Movements 3 to 7 times per week** | 9 (69.2%) | 7 (58.3%) | 16 (64.0%) |
| **Bowel Movements >7 times per week** | 4 (30.8%) | 5 (41.7%) | 9 (36.0%) |
| **Significant Medical History displayed by System Order Class – Preferred terms (SOC-PT)** | | | |
| **At least one significant medical history** | 1 (7.7%) | 2 (16.7%) | 3 (12.0%) |
| **Neoplasms: Benign, Malignant and Unspecified (Incl Cysts and Polyps)** | 0 | 1 (8.3%) | 1 (4.0%) |
| **Malignant Melanoma** | 0 | 1 (8.3%) | 1 (4.0%) |
| **Nervous System Disorders** | 0 | 1 (8.3%) | 1 (4.0%) |
| **Hydrocephalus** | 0 | 1 (8.3%) | 1 (4.0%) |
| **Respiratory Thoracic and Mediastinal Disorders** | 0 | 1 (8.3%) | 1 (4.0%) |
| **Asthma** | 0 | 1 (8.3%) | 1 (4.0%) |
| **Skin And Subcutaneous Tissue Disorders** | 0 | 1 (8.3%) | 1 (4.0%) |
| **Henoch-Schonlein Purpura** | 0 | 1 (8.3%) | 1 (4.0%) |
| **Surgical And Medical Procedures** | 1 (7.7%) | 1 (8.3%) | 2 (8.0%) |
| **Compliance with intake of Indomethacin (mean [SD] %)** | | | |
| **Compliance to dose** | 98.3 (4.9) | 100.0 (0.0) |  |
| **Treatment duration >80% compliant** | 100 | 100 |  |
